# Enhancing DNN Adversarial Robustness via Dual Stochasticity and Geometric Normalization

**DOI:** 10.3390/s25237398

**Published:** 2025-12-04

**Authors:** Xiang Wu, Gangtao Han

**Affiliations:** 1 School of Cyber Science and Engineering, Zhengzhou University, Zhengzhou 450001, China; wuxiang@gs.zzu.edu.cn; 2 School of Electrical and Information Engineering, Zhengzhou University, Zhengzhou 450001, China

**Keywords:** deep neural networks, adversarial attacks, adversarial robustness, model uncertainty

## Abstract

Deep neural networks (DNNs) have achieved remarkable progress across various domains, yet they remain highly vulnerable to adversarial attacks, which significantly hinder their deployment in safety-critical applications. While stochastic defenses have shown promise, most existing approaches rely on fixed noise injection and fail to account for the geometric stability of the decision space. To address these limitations, we introduce a novel framework, which named as Dual Stochasticity and Geometric Normalization (DSGN). Specifically, DSGN incorporates learnable, input-dependent Gaussian noise into both the feature representation and classifier weights, creating a dual-path stochastic modeling mechanism that captures multi-level predictive uncertainty. To enhance decision consistency, both noisy components are projected onto a unit hypersphere via ${𝓁}_{2}$ normalization, constraining the logit space and promoting angular margin separation. This design stabilizes both the representation and decision geometry, leading to more stable decision boundaries and improved robustness. We evaluate the effectiveness of DSGN on several benchmark datasets and CNNs. Our results indicate that DSGN achieves a robust accuracy improvement of approximately 1% to 6% over the state-of-the-arts baseline model under PGD and 1% to 17% improvement under AutoAttack, demonstrating its effectiveness in enhancing adversarial robustness while maintaining high clean accuracy.

## 1. Introduction

In recent years, deep neural networks (DNNs) have achieved significant success in tasks such as image classification [1], object detection [2,3], and natural language processing [4]. However, despite its excellent performance, DNN is still highly susceptible to adversarial samples—these carefully designed, almost imperceptible small perturbations to humans can cause the model to make erroneous or even dangerous predictions [5,6], posing significant challenges to its reliable deployment in safe and critical task scenarios. Even more concerning is that such vulnerabilities are not isolated phenomena. The comprehensive survey “Threatened Artificial Intelligence Agents: Key Security Challenges and Future Directions” [7] further highlights that adversarial attacks have become one of the main security risks faced by artificial intelligence systems, exacerbating concerns about their credibility and robustness.

Adversarial attacks are typically categorized into white-box and black-box paradigms. In white-box settings, attackers have full access to the model’s architecture, parameters, and gradients, allowing them to craft highly effective adversarial examples via backpropagation [5,8]. In contrast, black-box attacks estimate gradients via surrogate models or query feedback, yet still manage to generate transferable perturbations that can evade defenses [9]. These threats underscore the urgent need for robust and generalizable defense mechanisms.

A promising line of research introduces stochasticity into deep networks to improve adversarial robustness [10,11,12,13]. Instead of relying on fixed parameters, these approaches introduce randomness into the model, allowing it to better capture uncertainty and explore a local neighborhood in the parameter space. This idea is partly inspired by probabilistic modeling principles in variational autoencoders and Bayesian neural networks, where stochastic latent representations have been shown to improve uncertainty estimation and robustness under distributional variations. Theoretical studies also suggest that randomized models can provide stronger robustness guarantees than deterministic ones under certain conditions [14,15].

However, most existing randomization-based defenses adopt fixed noise distributions and operate at a single model level, which restricts their adaptability to input-dependent uncertainty and fails to capture multi-level stochastic interactions within the network [10,12]. Moreover, these methods often ignore the geometric structure of features and classifier weights, leading to poorly calibrated decision boundaries and reduced consistency between clean and adversarial predictions [16].

To solve these issues, this work introduces Dual Stochasticity and Geometric Normalization (DSGN), a novel defense framework that compellingly integrates a dual-path stochastic mechanism with geometric normalization to significantly enhance the adversarial robustness of deep networks. Specifically, DSGN injects learnable, input-dependent Gaussian noise into both feature embeddings and classifier weights, enabling data-driven uncertainty modeling across representational and decision levels. To explicitly stabilize the model geometry, we normalize the noisy features and weights onto the unit hypersphere, thereby enforcing consistent vector magnitudes across both spaces. This constrains the decision function to rely mainly on angular differences, which are inherently more robust to norm-bounded adversarial perturbations that typically attempt to exploit radial (magnitude) changes. By restricting radial variations, the normalization enforces smoother and more stable decision boundaries, enhancing overall adversarial robustness and training stability.

Unlike prior randomization defenses that rely on fixed noise or single-level injection, DSGN jointly models uncertainty in both representation and decision spaces with adaptive noise, and explicitly regularizes the geometry of features and classifier weights. Extensive experiments on multiple benchmarks and diverse attack settings demonstrate that DSGN consistently enhances adversarial robustness while maintaining high clean accuracy.

Our contributions are summarized as follows:

- We develop a dual-path stochastic mechanism that injects learnable, input-conditioned Gaussian noise into both feature embeddings and classifier weights, enabling joint uncertainty modeling across representational and decision levels.
- We introduce a geometric normalization strategy that projects noisy features and classifier weights onto a unit hypersphere, enhancing angular discriminability and improving decision consistency.
- We provide both theoretical and empirical analyses demonstrating how stochastic and geometric components jointly stabilize the logit geometry, yielding stronger adversarial robustness while maintaining high clean accuracy.

The rest of the article is organized as follows. Section 2 reviews essential concepts and techniques related to adversarial attacks, defense strategies. Section 3 introduces our proposed framework designed to enhance model robustness under adversarial conditions. In Section 4, we present a comprehensive set of experiments to evaluate and demonstrate the effectiveness of our approach. Finally, Section 5 offers concluding remarks and discusses potential future directions.

## 2. Related Work

### 2.1. Adversarial Attacks

DNNs have demonstrated remarkable performance in various machine learning applications, such as image recognition, natural language processing, and autonomous driving. However, despite their success, DNNs remain vulnerable to adversarial attacks. These attacks involve adding small, carefully crafted perturbations to input samples that cause DNNs to make incorrect predictions. The vulnerability of DNNs to imperceptible perturbations was first discovered by Szegedy et al. [17], sparking significant interest in the study of adversarial attacks. Since then, a wide range of attack methods have been proposed [5,18,19,20], which can be broadly categorized into two types: white-box attacks and black-box attacks, depending on the adversary’s level of knowledge about the target model.

White-box attacks assume full access to the target model, including its architecture, parameters, and gradients. This enables precise, gradient-based adversarial example generation. For example, the Fast Gradient Sign Method (FGSM) [5] perturbs the input in the direction of the loss gradient. Its iterative variant, Iterative FGSM (I-FGSM) [18], applies multiple small-step updates to produce stronger perturbations. Projected Gradient Descent (PGD) [8], a widely adopted attack, extends I-FGSM by incorporating a projection step to ensure that the perturbed sample remains within the allowed ${𝓁}_{p}$-norm constraint, thereby enhancing attack strength and stability. The Carlini & Wagner (C&W) attack [19] formulates adversarial generation as an optimization problem, aiming to minimize perturbation magnitude while enforcing misclassification with high confidence. More recently, AutoAttack [20] has emerged as a robust and reliable evaluation suite. It is a parameter-free ensemble of attacks, combining complementary strategies such as APGD and the Square Attack [21] to produce a comprehensive robustness assessment.

Black-box attacks, in contrast, assume no internal access to the model and rely solely on input–output behavior. These attacks are typically divided into two categories: query-based and transfer-based. Query-based attacks [21,22,23] iteratively generate adversarial examples by probing the model with carefully designed inputs and observing the outputs. Although they can be effective, these methods often come with substantial computational costs, as they require a large number of queries. Transfer-based attacks [24,25,26] exploit the transferability property of adversarial examples, where inputs generated to fool a surrogate model can also mislead an unseen target model. Despite their computational efficiency, the effectiveness of transfer-based methods often diminishes when the target model is robust or architecturally different from the surrogate model. To improve transferability, recent research has proposed strategies such as input diversity [27], feature alignment between models [28] and the use of ensemble surrogates [29] to better approximate the decision boundaries of the target model. In summary, the growing sophistication of adversarial attacks, summarized in Table 1, continues to challenge the reliability of deep learning systems. This underscores the importance of developing robust defense strategies and standardized evaluation protocols to assess model resilience under various adversarial threat models.

### 2.2. Adversarial Defense

Defending against adversarial attacks has become an increasingly critical problem, drawing substantial attention from the research community [30]. Among various defense mechanisms, adversarial training is widely regarded as one of the most effective and commonly adopted proactive defense strategies [8,30]. This method enhances model robustness by explicitly incorporating adversarial examples into the training process. By optimizing the model to correctly classify both clean and adversarial inputs, adversarial training encourages the learning of smoother loss landscapes and more robust feature representations, thereby improving resistance to worst-case perturbations.

Although adversarial training significantly improves robustness, it often introduces substantial computational overhead and leads to a reduction in clean-data accuracy  [14,15]. To address these limitations, numerous improved variants have been developed. For instance, Liu et al. [31] proposed Collaborative Adversarial Training (CAT), an ensemble-based method where multiple models are trained under diverse adversarial objectives, enabling mutual knowledge transfer and enhanced robustness. Yin et al. [32] introduced Logit-Oriented Adversarial Training (LOAT), which incorporated Fisher-Rao norm regularization to refine the decision boundaries, effectively mitigated the robustness–accuracy trade-off. More recently, Bai et al. [33] presented Wasserstein distributional adversarial training (WAT), a Wasserstein-based adversarial training method grounded in optimal transport theory, enabling better alignment between clean and adversarial distributions to improve generalization across threat models.

Beyond deterministic defenses, randomization-based methods have emerged as a promising alternative approach. These techniques introduced stochasticity at various stages of training or inference to obscure gradients and hinder adversarial optimization. For example, Parametric Noise Injection (PNI) [34] introduced learnable Gaussian noise into weights or activations, effectively enhancing model robustness while preserving its representational capacity. Jin et al. [35] proposed weight-level noise guided by first-order Taylor approximations, effectively flattening the loss landscape to suppress adversarial sensitivity. Liu et al. [36] designed a selective neuron-level noise injection strategy that targets non-critical pathways, balancing robustness with computational cost.

Recent works further reinforce the potential of randomized defenses. Pinot et al. [37] demonstrated the limitations of deterministic classifiers and showed that stochastic ensembles could achieve improved adversarial robustness under specific conditions. Subsequently, certified defenses such as randomized smoothing [38] offered probabilistic robustness guarantees by leveraging the expected prediction of noise-injected models, marking a rigorous advancement in verifiable defense mechanisms.

Despite significant advancements, randomization-based defenses encounter several key challenges. First, the incorporation of stochasticity often destabilizes training and slows convergence, particularly when applied to large-scale datasets. Furthermore, the lack of a unified theoretical framework for determining critical noise parameters results in a reliance on empirical tuning and heuristics. Additionally, current methods exhibit limited scalability and generalization across diverse architectures and data domains. Consequently, understanding the complex interplay between randomness, optimization dynamics, and generalization remains an open problem and a dynamic area of ongoing research.

## 3. Methodology

**Figure 1:**
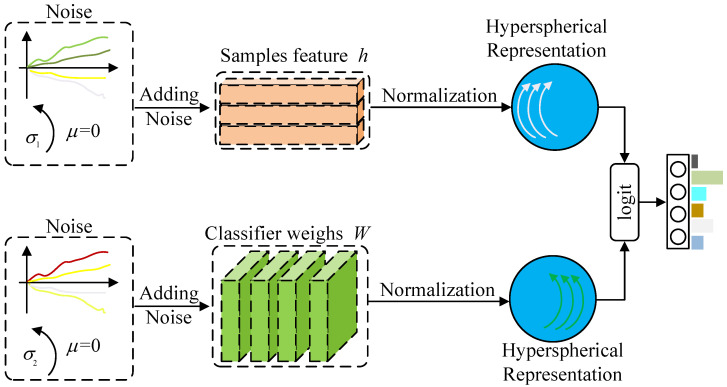
Overview of the DSGN framework. To model uncertainty, the DSGN framework injects zero-mean Gaussian noise into both feature representations and classifier weights. The noise is modeled as a zero-mean Gaussian distribution with a variance that increases progressively over the course of training. The resulting noisy features and classifier weights are then normalized via ${𝓁}_{2}$-normalization to produce unit vectors, which are used in the final logit computation.

As illustrated in Figure 1, the proposed DSGN introduces learnable stochasticity into both feature representations and classifier weights, followed by geometric normalization on a unit hypersphere. This section provides the implementation details of the DSGN framework.

### 3.1. Random Feature

Our approach injects stochastic noise directly into the feature representations at the penultimate layer of the feature extractor. The key idea is to progressively increase the noise variance throughout training, starting with small stochastic noises and gradually amplifying them. This progressive noise injection acts as an implicit regularizer, encouraging the model to learn decision boundaries that are robust to both adversarial attack.

We provide a theoretical framework that explains how stochastic noise induces implicit regularization against input perturbations. Given a mini-batch of training samples $B={\left\{(x_{i},y_{i})\right\}}_{i=1}^{\left|B\right|}$, we inject stochastic noise into the activations $h\in R^{D}$ at the penultimate layer of the neural network to improve model robustness. Specifically, When Gaussian noise ϵ, with zero mean and variance $\Sigma_{1}$, is introduced, the activation is rewritten as

(1) $$\tilde{h}=h+\epsilon .$$

Let L(x,y) denote the standard loss on clean input. After noise injection, the modified loss becomes

(2) $$L\left(x,\epsilon ,y\right)=L\left(\tilde{h},y\right).$$

Using the exponential shift operator identity

(3) $$f\left(z+\delta \right)=e^{\delta^{⊤}\nabla_{z}}f\left(z\right),$$

We can compute the average batch loss by

(4) $$L_{avg}=\frac{1}{\left|B\right|}\sum_{(x,y)\in B}e^{\epsilon^{⊤}\nabla_{h}}L\left(x,y\right).$$

Expanding the exponential operator with Taylor series, we obtain

(5) $$L_{avg}=L_{0}+\sum_{k=1}^{\infty }R_{k},$$

where $L_{0}$ denotes the average loss on clean inputs, $R_{k}$ are batch-averaged derivatives of the loss function with respect to the preactivations at the noise-injected layer, and we have

(6) $$R_{k}=\frac{1}{k!}\cdot \frac{1}{\left|B\right|}\sum_{(x,y)\in B}{\left(\epsilon^{⊤}\nabla_{h}\right)}^{k}L\left(x,y\right).$$

For zero-mean Gaussian noise, the first two terms can be simplified as

(7) $$R_{1}=\epsilon^{⊤}\nabla_{h}L\left(x,y\right),$$

(8) $$R_{2}=\frac{1}{2}\epsilon^{⊤}\nabla_{h}^{2}L\left(x,y\right)\;\varepsilon ,$$

where $\nabla_{h}L\left(x,y\right)$ and $\nabla_{h}^{2}L\left(x,y\right)$ denote the gradient and Hessian of the loss with respect to the activations at the penultimate layer, correspondingly.

When the noise level is small, the first-order term $R_{1}$ becomes the dominant effect, serving as a mild regularizer that stabilizes gradient fluctuations and helps mitigate overfitting. As the noise level increases, the second-order term $R_{2}$ becomes more influential, penalizing directions in the loss landscape with high curvature. This progressive effect leads to a smoother optimization surface, thereby enhancing the model’s robustness to both adversarial attacks.

### 3.2. Random Weight

To improve the model’s generalization and robustness, we introduce stochasticity at the level of the classifier’s weight matrix. Specifically, instead of using a deterministic weight matrix $W\in R^{C\times D}$, we introduce noise E, each element following zero-mean Gaussian distribution, and variance $\Sigma_{2}$ to randomize the weights. This noisy weight matrix, denoted as $\tilde{W}$, is expressed as

(9) $$\tilde{W}=W+E,$$

Let the logit output before softmax be defined as z=Wh. After adding the noise, the noisy logit becomes

(10) $$\tilde{z}=\tilde{W}h=\left(W+E\right)h=z+Eh.$$

Let L(z,y) denote the standard loss computed from logits and ground truth labels. The loss becomes

(11) $$L\left(\tilde{z},y\right)=L\left(z+Eh,y\right).$$

Using the exponential shift operator in the logit space:

(12) $$f\left(z+\delta \right)=e^{\delta^{⊤}\nabla_{z}}f\left(z\right),$$

We can expand the loss as

(13) $$L=L_{0}+\sum_{k=1}^{\infty }\frac{1}{k!}{\left(Eh\right)}^{⊤}\nabla_{z}^{k}L\left(z,y\right),$$

where $L_{0}=L\left(z,y\right)$ is the clean loss, and higher-order terms represent the influence of weight noise.

Taking the expectation over the Gaussian noise, the first-order term vanishes (due to symmetry), and the leading contribution comes from the second-order term:

(14) $$E\left[L\right]\approx L_{0}+\frac{1}{2}E\left[{\left(Eh\right)}^{⊤}\nabla_{z}^{2}L\left(z,y\right)\left(Eh\right)\right].$$

This simplifies to

(15) $$E\left[L\right]\approx L_{0}+\frac{1}{2}\cdot \mathrm{Tr}\left(h^{⊤}\Sigma_{2}h\cdot \nabla_{z}^{2}L\left(z,y\right)\right).$$

This shows that weight noise introduces an implicit curvature-aware regularizer that penalizes the sensitivity of the loss with respect to logits, scaled by the energy of the input features. Similar to feature noise, the second-order term acts as a smoothing constraint on the loss landscape, but now in the weight (parameter) space. As the model is trained with increasing noise levels, it learns to avoid regions with sharp logit sensitivity, resulting in improved generalization and robustness.

### 3.3. Hyperspherical Geometry via Dual Normalization

To reduce instability caused by magnitude variations and enforce geometric consistency, we apply ${𝓁}_{2}$ normalization to both feature vectors and classifier weights. Given a feature vector $h\in R^{D}$, we normalize it to lie on the unit hypersphere:

(16) $$h_{\mathrm{norm}}=\frac{h}{{∥h∥}_{2}}.$$

Similarly, each class weight vector $w_{c}\in R^{D}$ is normalized as

(17) $$w_{c,\mathrm{norm}}=\frac{w_{c}}{∥w_{c}{∥}_{2}},\;c=1,\ldots ,C.$$

This transforms the conventional linear classifier into a cosine classifier:

(18) $$f_{c}\left(h\right)=w_{c,\mathrm{norm}}h_{\mathrm{norm}}=\cos\left(\theta_{c}\right),$$

where $\theta_{c}$ is the angle between the normalized feature and class weight. The model predicts based solely on angular similarity, discarding scale information.

Normalization simultaneously removes magnitude bias in both features and weights and induces an angular decision boundary geometry, providing several advantages. Without normalization, the classifier is sensitive to feature norm ∥h∥, biasing training toward easy examples with large norms; normalization enforces fair gradient flow across samples of all difficulty levels. Weight normalization restricts all classes to lie on a hypersphere, ensuring uniform decision geometry and reducing inter-class bias caused by class imbalance or perturbations. Furthermore, under normalization, adversarial examples must induce angular deviation to succeed, making magnitude-based attacks ineffective, as the logits are only influenced by the angular relationship between the feature and the weight vectors.

Considering a small perturbation δ added to h, the normalized adversarial feature is

(19) $$h_{\mathrm{adv}}=\frac{h+\delta }{{∥h+\delta ∥}_{2}}.$$

Assuming ${∥\delta ∥}_{2}\ll {∥h∥}_{2}$, a first-order approximation yields

(20) $$\cos\left(\theta_{c}+\Delta \theta_{c}\right)\approx \cos\left(\theta_{c}\right)-\sin\left(\theta_{c}\right)\cdot \Delta \theta_{c},$$

which bounds logit changes by the angular shift $\Delta \theta_{c}$, making the model more resistant to norm-based adversarial manipulation.

### 3.4. Overall Loss

As previously mentioned, enhancing uncertainty estimation during training is essential. Directly specifying parameter variances is infeasible because the required uncertainty depends on the model architecture and dataset, which are typically unknown. To address this, we initialize the Gaussian variance with a non-informative uniform prior. Throughout training, the variance gradually increases in small increments. Moreover, the variance remains bounded because its gradient is inversely proportional to the variance. As the variance increases, the gradient magnitude decreases, preventing further escalation.

The overall loss is formulated as

(21) $$L=L_{\mathrm{CE}}-\sum_{i=1}^{D}\ln\left(\sigma_{1,i}\right)-\sum_{j=1}^{C}\sum_{k=1}^{D}\ln\left(\sigma_{2,jk}\right),$$

where $L_{\mathrm{CE}}$ denotes the standard cross-entropy loss. Here, the feature noise variance $\sigma_{1}$ is parameterized per element of the feature vectore of size *D*, and the weight noise variance $\sigma_{2}$ is parameterized per element of the classifier weight matrix of size C×D. The negative log terms serve as entropy regularizers on the noise variances, preventing them from growing excessively. The training procedure of the proposed DSGN method is summarized in Algorithm 1.
**Algorithm 1** Training procedure of the proposed DSGN**Require:** Mini-batch samples (x,y); classifier weights $W\in R^{C\times D}$, bias b; the hyper-parameter $\lambda_{1},\lambda_{2}>0$**Ensure:** Updated model parameters θ1:**for** each training epoch **do**  2:   **for** each mini-batch **do**    3:   Extract features from backbone: $h=\mathrm{FeatureExtractor}\left(x\right)\in R^{D}$    4:   Sample feature noise: $\epsilon ∼N(0,\Sigma_{1})$    5:   Inject noise into features: $\tilde{h}\leftarrow h+\lambda_{1}\epsilon$    6:   Normalize feature vector: $h_{\mathrm{norm}}\leftarrow \frac{\tilde{h}}{∥\tilde{h}{∥}_{2}}$    7:   Sample noise for classifier weights: $E∼N(0,\Sigma_{2})$    8:   Inject noise into weights: $\tilde{W}\leftarrow W+\lambda_{2}\;E$    9:   Normalize each weight vector (per class): $w_{c,\mathrm{norm}}\leftarrow \frac{{\tilde{w}}_{c}}{∥{\tilde{w}}_{c}{∥}_{2}},\forall c=1,\ldots ,C$    10:   Compute logits: $z\leftarrow W_{\mathrm{norm}}h_{\mathrm{norm}}+b$         11:   Compute loss:    $$\;\;L_{\mathrm{total}}=L_{\mathrm{CE}}\left(z,y\right)-\sum_{i=1}^{D}\ln\left(\sigma_{1,i}\right)-\sum_{c=1}^{C}\sum_{d=1}^{D}\ln\left(\sigma_{2,cd}\right)\;\;$$12:   Update model parameters via backpropagation  13:    **end for**14:**end for**

## 4. Experiments

### 4.1. Experiment Setup

**Datasets:** We evaluate our method on seven widely used benchmark datasets: MNIST [39], SVHN [40], CIFAR-10 [41], CIFAR-100 [41], Tiny ImageNet [40], Imagenette [42], and ImageNet [43]. MNIST consists of 60,000 training and 10,000 testing grayscale images of handwritten digits, each with a resolution of 28×28. SVHN contains 73,257 training and 26,032 testing RGB images of street-view house numbers at 32×32 resolution, spanning 10 digit classes. CIFAR-10 and CIFAR-100 both comprise 32×32 natural color images, with CIFAR-10 covering 10 general object categories, while CIFAR-100 offers a more challenging setting with 100 fine-grained classes. Tiny ImageNet contains 200 categories, each with 500 training images resized to 64×64 resolution. Imagenette is a curated subset of ImageNet consisting of approximately 13,000 images from 10 selected classes, with all images uniformly resized to 160×160. ImageNet (ILSVRC 2012) is a large-scale benchmark dataset comprising over 1.2 million labeled training images and 50,000 validation samples across 1000 object categories, providing a realistic and challenging evaluation setting for high-capacity models.

**Adversarial Attacks:** To evaluate the robustness of the proposed framework, we test it against a range of adversarial attacks, including FGSM [5], PGD [8], C&W [19], MIFGSM [29], DeepFool [44], and AutoAttack [45]. All attacks are implemented following widely adopted settings. For FGSM, PGD, MIFGSM, and AutoAttack, the perturbation limit is ϵ = 8/255. The step size for PGD and MIFGSM is 2/255, with 20 and 5 steps, respectively. For the C&W attack, we set the learning rate to 0.01 and run it for 1000 steps. DeepFool uses 50 steps and an overshoot of 0.02. On the ImageNet dataset, the perturbation bound is reduced to ϵ = 4/255, with attack steps set to 10 and 50.

**Baseline:** To ensure a fair and comprehensive evaluation, we compare our method with a diverse set of baselines, including both randomized and non-randomized adversarial defenses. For randomized defenses, We consider Additive Noise [13] and its multiplicative noise injection variant, as well as Random Bits [46], RPF [47], CTRW [48], and RN [36] as baseline methods. For non-randomized baselines, we include several adversarial training-based methods such as RobustWRN [49], AWP [50], SAT [51], LLR [52], and RobNet [53]. These baselines cover a broad range of defense strategies and enable rigorous comparison across different threat models.

### 4.2. Results on CIFAR

To demonstrate the effectiveness of our proposed method, we first performed evaluations under six different adversarial attacks. Beyond the standard baseline of a deterministic classifier with adversarial training (denoted as AT), we included two additional noise-based baselines: additive noise injection and a stronger baseline, multiplicative noise injection, which simply fuses the feature maps via multiplying the noise. Additive noise is sampled from a standard Gaussian distribution N(0, 1), while multiplicative noise is sampled from N(1, 1). Furthermore, we also compared our results with the latest defense algorithms, including RPF, CTRW and RN. Although these noise-based baselines are simple, they can achieve satisfactory adversarial robustness within our defense scheme. The results are summarized in Table 2.

On the CIFAR-10 dataset, the analysis of noise injection methods reveals clear gains over the AT baseline. Additive Noise achieves a 5.45% increase in PGD^20^ robustness over AT (from 52.16% to 57.61%), while Multiplicative Noise further improves this to 59.49% under PGD^20^ and 63.78% under AutoAttack, accompanied by modest gains in clean accuracy. The RPF method, leveraging geometric constraints, elevates PGD^20^ robustness to 61.27% and AutoAttack accuracy to 64.38%, signaling the advantage of structured randomness. The CTRW method achieves a significant PGD^20^ robustness of 69.48% and an AutoAttack accuracy of 73.56%, marking an important advance in robustness. The RN method demonstrates strong resistance to multi-step attacks, achieving 74.55% robustness against PGD^20^. Our proposed approach, DSGN, consistently surpasses all prior competitors. DSGN achieves a superior clean accuracy of 92.21% and attains 80.43% robust accuracy against PGD^20^, representing a 5.88% improvement over the strongest prior competitor RN (74.55%). Furthermore, under the AutoAttack benchmark, DSGN achieves an impressive 84.52% robustness, which is a remarkable 20.14% improvement compared to RPF (64.38%). Crucially, our model uniquely maintains over 80% robustness consistently across multiple attack types, including FGSM (82.32%), PGD^20^ (80.43%), and MIFGSM (80.54%), while simultaneously maintaining superior clean accuracy, effectively mitigating the common trade-off between robustness and accuracy.

The performance disparity becomes even more pronounced on the more challenging CIFAR-100 dataset, highlighting the limitations of prior work in high-complexity settings. The AT baseline struggles significantly, achieving only 28.71% robustness under PGD^20^ and 24.48% under AutoAttack. Both Additive and Multiplicative noise injection methods yield moderate improvements, yet their AutoAttack accuracies remain below 39%. The RPF method pushes the robustness envelope further, reaching 42.88% AutoAttack accuracy. The CTRW method achieves 42.01% for PGD^20^ and 45.58% for AutoAttack. The RN method provides a PGD^20^ robustness of 47.70%, but its AutoAttack robustness drops to 39.01%. However, our method, DSGN, sets a new standard with 56.34% precision under AutoAttack and 48.23% robustness under PGD^20^, representing a significant improvement of over 10% compared to RPF under AutoAttack. Equally important is the clean accuracy, where DSGN attains 67.71%, substantially outperforming all prior baselines, such as RPF (56.88%). This result underscores our model’s ability to circumvent the traditional robustness–accuracy trade-off even on complex datasets with higher class cardinality. Collectively, these findings robustly validate the superiority of our DSGN method across all evaluated metrics and datasets, demonstrating its effectiveness in achieving state-of-the-art performance in both clean accuracy and adversarial robustness.

We further evaluate the adversarial robustness of our method on CIFAR-10 using the WideResNet-34-10 architecture, and compare it against a variety of randomized baselines, as summarized in Table 3. Consistent with the observations on ResNet-18, our method outperforms all competing baselines across four commonly used adversarial attacks. Compared with the AT, our method achieves significantly higher robust accuracy, with a 27.18% improvement under PGD^20^ and a 29.68% gain under AutoAttack. Similarly, when compared to the additive noise injection, which achieves 60.55% accuracy under AutoAttack, our method improves the robustness by 21.37%. This suggests that our approach provides more stable and effective defense even under stronger attack settings. In addition, our method also surpasses advanced randomized defenses such as Random Bits, Multiplicative Noise, RPF, and CTRW, demonstrating both higher clean and robust accuracy. For instance, compared to CTRW, which achieves 74.23% robust accuracy under AutoAttack, our method improves robust accuracy by 7.69%. Furthermore, even against the most competitive recent baseline RN, our method achieves a consistent improvement across all attack scenarios. Specifically, we observe a 1.36% improvement under AutoAttack and a 1.25% boost under PGD^20^.

### 4.3. Evaluate with Stronger Attacks

**Figure 2:**
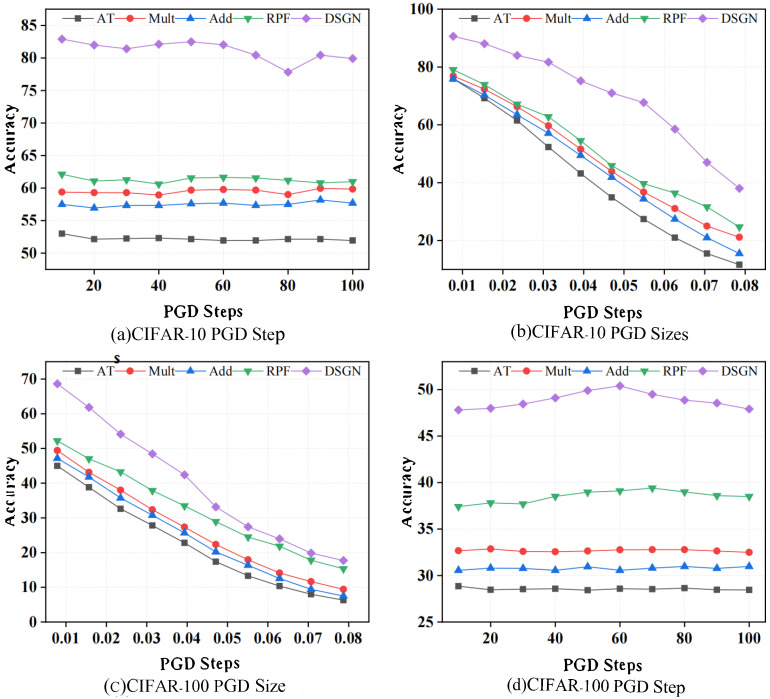
The evaluation of stronger PGD attacks with ResNet-18 on CIFAR-10 and CIFAR-100.

In addition to the standard evaluation of adversarial robustness presented in Table 2, we further evaluate the performance of various defense methods under stronger attack settings on CIFAR-10 and CIFAR-100, as shown in Figure 2. We mainly consider two scenarios: increasing the number of PGD attack steps (from ${\mathrm{PGD}}^{10}\;\mathrm{to}\;{\mathrm{PGD}}^{100}$) and increasing the magnitude of the perturbation ϵ (with ϵ∈[2/255,20/255]). We compare our method with Overfit (denoted as AT), RPF (denoted as RPF), Additive Noise (denoted as Add), and Multiplicative Noise (denoted as Mult). In the scenario with increased PGD steps, Figure 2a,c demonstrate the robustness of different methods on CIFAR-10 and CIFAR-100, respectively. Unlike the deterministic adversarial training baseline, randomized methods exhibit stronger resilience against the increasing number of attack iterations. Among these, Ours consistently achieves the highest robust accuracy, maintaining 80.0% on CIFAR-10 and 48.0% on CIFAR-100 under PGD_100_. In contrast, RPF attains 60.6% and 39.0%, while Additive and Multiplicative noise injection methods perform even lower.

Although all methods experience performance degradation as the perturbation size ϵ increases, our method consistently maintains the highest robust accuracy across all settings, as shown in Figure 2b,d. On CIFAR-10, our method achieves 58.4% robust accuracy at ϵ = 0.06, clearly outperforming RPF (36.2%) as well as additive and multiplicative noise baselines. Even under stronger perturbations (e.g., ϵ = 20/255), our method still reaches 36.6% accuracy, the best among all evaluated methods. A similar trend is observed on CIFAR-100: at ϵ = 20/255, our method maintains 18.6% robust accuracy, surpassing RPF (16.6%) and other baselines.

### 4.4. State-of-the-Art Comparison

We also compare our proposed DSGN with leading defense methods to highlight its effectiveness. The evaluation is conducted on two widely used benchmarks: WideResNet-34-10 for CIFAR-10 and ResNet-50 for ImageNet. The results are summarized in Table 4. Specifically, we report robust accuracies on CIFAR-10 under PGD^20^ and AA, and on ImageNet under PGD^10^ and PGD^50^. Our method consistently outperforms all existing approaches across both datasets and evaluation settings. On CIFAR-10, it achieves robust accuracies of 83.31% under PGD^20^ and 84.92% under AA, surpassing the previously strongest baseline RN by 0.32% and 16.22%, respectively. On ImageNet, our method attains robust accuracies of 74.26% and 72.27% under PGD^10^ and PGD^50^, representing clear improvements over both CTRW and RN. In particular, our method outperforms RPF by 17.70% under PGD^10^ and 16.86% under PGD^50^. These results demonstrate the strong robustness and scalability of our method, especially under more challenging attack scenarios and on large-scale datasets.

### 4.5. Inspection of Generalization

We conduct comprehensive experiments on diverse datasets and network architectures to assess the generalization capability of the proposed method. The experimental results are summarized in Table 5 and Table 6. In Table 5, we focus on evaluating the robustness of our method across six widely used image classification datasets: MNIST, SVHN, CIFAR-10, CIFAR-100, Tiny-ImageNet and Imagenette. All experiments are conducted using ResNet-18 as the backbone, except for MNIST, which uses LeNet. We report accuracy under clean, FGSM, and PGD^20^ attack settings. Compared to the baseline (no defense), our method achieves significantly improved robustness across all datasets, particularly under strong PGD attacks. For instance, on SVHN and CIFAR-10, our method achieves over 80% accuracy under PGD^20^ attacks, while the baseline model suffers severe degradation. These results indicate that our method maintains strong generalization capability across datasets of varying complexity and scale. In Table 6, we investigate the impact of different network architectures on our method. We evaluate the performance of our method on several mainstream models, including VGG19, GoogLeNet, DenseNet121, and various ResNet variants (ResNet-32/44/56). As shown in the results, our method consistently outperforms the baseline under adversarial settings for all architectures. Notably, our method maintains high robustness even on deeper networks such as ResNet-56, achieving 69.1% accuracy under PGD^20^, compared to only 1.2% for the no defense. This suggests that our method generalizes effectively across networks of varying depths and design paradigms. In summary, we evaluate the generalization ability of our method from two perspectives: datasets and architectures. For datasets, our method demonstrates strong robustness across a wide range of data distributions. For architectures, we confirm that our method adapts well to different network types, as well as varying widths and depths. These results validate that our method is a highly generalizable defense method for adversarial robustness.

### 4.6. TSNE Visualization

**Figure 3:**
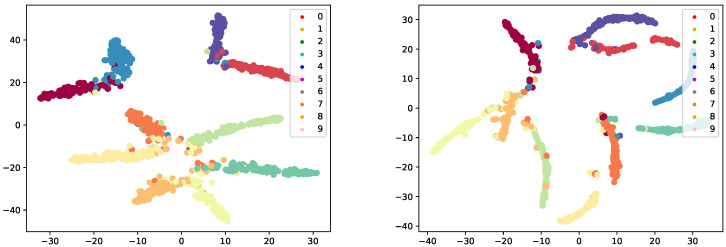
t-SNE visualization of classification results on CIFAR-10 trained with ResNet-18. No defense (**Left**) DSGN (**Right**).

We visualize the classification results on the CIFAR-10 dataset using a ResNet-18 model (Figure 3). In our experiments, we sampled 1000 data points and observed that DSGN effectively enhances intra-class compactness while increasing inter-class separability. This indicates that our method maintains a high level of uncertainty and adaptively learns deeper feature representations. Such uncertainty helps the network avoid local minima and explore global optima, ultimately boosting both robustness and classification accuracy.

### 4.7. Inspection of Gradient Obfuscation

Athalye et al. [55] pointed out that certain stochastic defense methods merely obscure gradient information to create the illusion of improved robustness, and as such, are considered “false defenses.” While these methods may temporarily impede gradient-based attacks, they are ultimately vulnerable to targeted attacks. To validate the list proposed by Athalye, we conduct a series of experiments following the experimental setup of Jeddie et al. [56] to evaluate these defenses. To verify that our proposed approach does not rely on gradient obfuscation, we conduct a series of diagnostic evaluations following the criteria proposed by Athalye and in accordance with the experimental protocol of Jeddi et al. [56].

#### 4.7.1. Criterion 1: One-Step Attacks Outperform Iterative Attacks

**Refutation:** It is well established that PGD attack is an iterative method, while the FGSM constitutes a single-step variant. As observed in Table 2 and Table 3, the DSGN model consistently attains higher accuracy against FGSM attacks compared with PGD attacks. This empirical evidence confirms that iterative attacks are indeed more potent than one-step methods, thereby refuting the characteristic behavior of gradient-obfuscated defenses.

#### 4.7.2. Criterion 2: Black-Box Attacks Outperform White-Box Attacks

**Refutation:** From Table 2 and Table 7, the white-box PGD attack demonstrates significantly stronger adversarial effectiveness than the 1- and 2-pixel black-box attacks. This outcome indicates that the defense remains vulnerable under full gradient access, reaffirming that the observed robustness does not stem from gradient masking or stochastic shielding.

#### 4.7.3. Criterion 3: Unbounded Attacks Fail to Achieve Complete Success

**Refutation:** In accordance with He et al. [34], we progressively increased the perturbation bound during evaluation. As shown in Figure 2 (left), the classification accuracy converges to nearly 0% under unbounded perturbations. This behavior is consistent with standard, non-obfuscated models, suggesting that our defense does not rely on deceptive gradient properties.

#### 4.7.4. Criterion 4: Random Sampling Can Independently Discover Adversarial Examples

**Figure 4:**
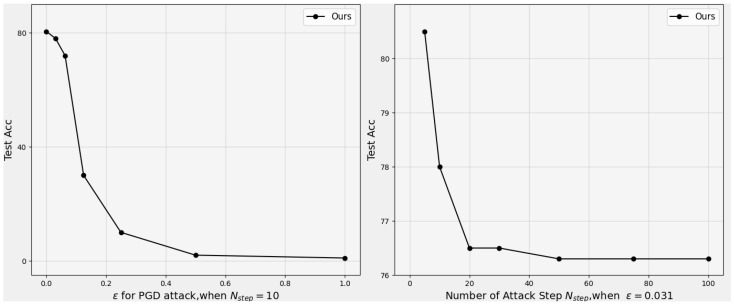
On the CIFAR-10 test set, the perturbed-data accuracy of ResNet-18 under PGD attack: (**Left**) versus attack bound ϵ, and (**Right**) versus the number of attack steps $N_{\mathrm{step}}$.

**Refutation:** As discussed by He et al. [34], random sampling should only succeed when gradient-based attacks fail. However, Figure 4 (right) clearly illustrates that as the perturbation bound increases, the proposed method remains susceptible to gradient-based attacks such as PGD. Therefore, the model’s robustness is not a byproduct of stochastic randomness or gradient discontinuities.

#### 4.7.5. Criterion 5: Increasing the Distortion Bound Does Not Improve the Attack Success Rate

**Refutation:** The empirical trend depicted in Figure 4 (left) shows that larger distortion bounds consistently lead to higher attack success rates (i.e., lower classification accuracy). This monotonic degradation aligns with the theoretical expectations of adversarial robustness under increasing perturbation magnitude, and further invalidates the possibility of gradient obfuscation.

#### 4.7.6. EOT-Based Evaluation

Athalye [55] also emphasized that defenses based on stochastic transformations are ineffective when gradients are estimated through the Expectation over Transformation (EOT) framework. Following Jeddi et al. [56] and Pinot et al. [57], we employ a Monte Carlo approximation of the gradient expectation over 80 random transformations on the CIFAR-10 dataset using a ResNet-18 backbone.

The results reveal that RPF, CTRW and RN achieve robustness levels of 59.32%, 66.74%, and 71.35%, respectively, while the proposed DSGN attains 78.32% robustness—substantially surpassing competing methods. This demonstrates that the proposed DSGN mechanism provides genuine robustness rather than relying on gradient obfuscation. To ensure gradient stability, we further apply 15 Monte Carlo samples during the testing phase, which produces consistent and reproducible performance across multiple evaluations.

### 4.8. Ablation Study

#### 4.8.1. The Effectiveness of Our Proposed Method

**Figure 5:**
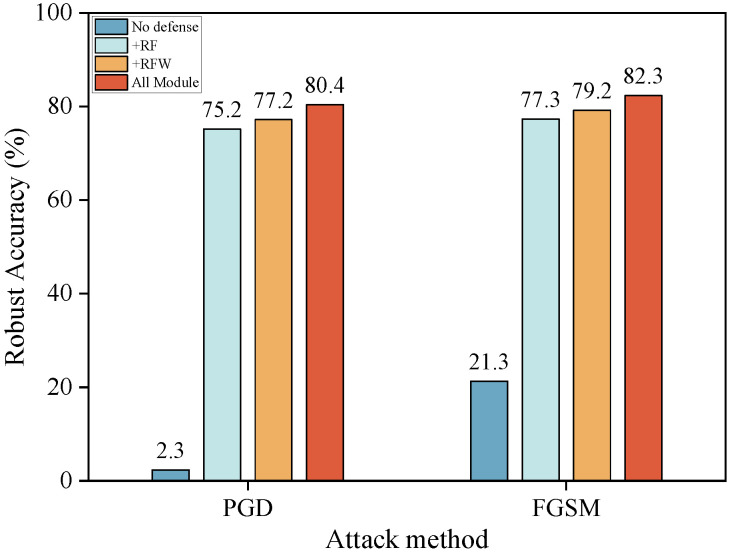
Ablation study of DSGN components.

To evaluate the effectiveness and necessity of each core component of the proposed DSGN framework, we conduct a detailed ablation study on the CIFAR-10 dataset using ResNet-18 backbone under both FGSM and PGD attack settings. As illustrated in Figure 5, we progressively integrate the three core components of DSGN: Random Feature, Random Weight, and Normalize of both feature and weight, to assess their individual and combined contributions to adversarial robustness.The baseline model, lacking any defense, exhibits extreme vulnerability, achieving only 21.3% and 2.3% robust accuracy against FGSM and PGD, respectively. Introducing the Random Feature (+RF) module yields the most substantial initial robustness gain, increasing accuracy to 77.3% (FGSM) and 75.2% (PGD). This highlights its critical role in learning perturbation-invariant feature representations. Subsequently, the addition of the Random Weight (+RFW) module further enhances robustness to 79.2% (FGSM) and 77.2% (PGD), confirming that stochasticity in the weight space improves generalization across diverse adversarial directions. Finally, the integration of all modules (All Module), including feature and weight normalization, achieves the best performance with 82.3% robust accuracy against FGSM and 80.4% against PGD. The final gain of approximately 3% is crucial because, in the highly competitive regime of adversarial defense against strong attacks like PGD, such incremental improvements are difficult to obtain and indicate that the normalization mechanism, by enforcing a geometric regularization that promotes inter-class separation on a hypersphere, is necessary to reach the highest level of adversarial resilience for the DSGN framework.

#### 4.8.2. Sensitivity of Hyper-Parameter

**Figure 6:**
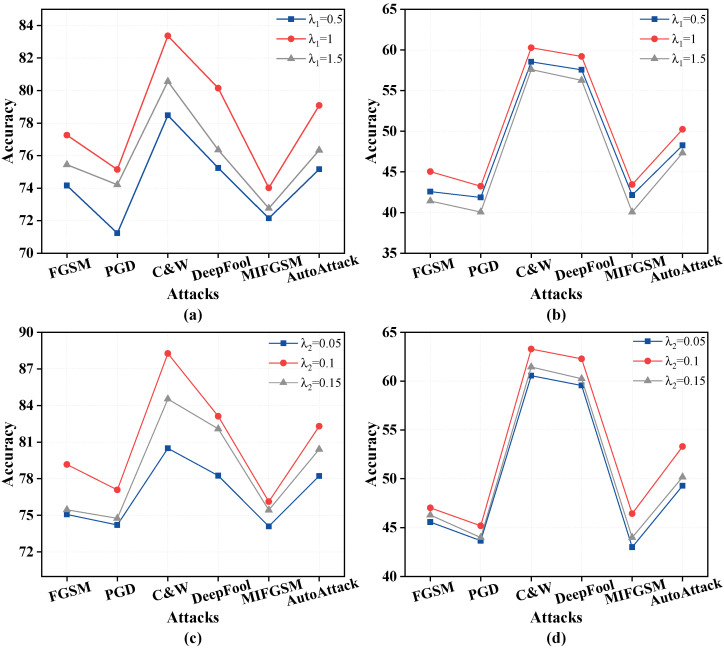
The evaluation of stronger PGD attacks with ResNet-18 on CIFAR-10 and CIFAR-100. (**a**) Impact of $\lambda_{1}$ on experimental results using CIFAR-10; (**b**) Impact of $\lambda_{1}$ on experimental results using CIFAR-100; (**c**) Impact of $\lambda_{2}$ on experimental results using CIFAR-10; (**d**) Impact of $\lambda_{2}$ on experimental results using CIFAR-100.

To investigate the effect of the hyperparameters $\lambda_{1}$ and $\lambda_{2}$ on the experimental results, we conducted an ablation study, with the results shown in Figure 6. Specifically, Figure 6a,c display the results on the CIFAR-10 dataset using the ResNet-18 model, while Figure 6b,d show the results on the CIFAR-100 dataset using the ResNet-18 model. First, we explored the impact of $\lambda_{1}$ on the performance of random features. As shown in Figure 6a,b, the optimal value was achieved when $\lambda_{1}=1$. Subsequently, with $\lambda_{1}$ fixed, we investigated the effect of $\lambda_{2}$ on eight randomness. The results indicate that the best performance was obtained when $\lambda_{2}=0.1$. Furthermore, we can observe that weights are more sensitive to noise compared to features.

## 5. Conclusions and Future Work

In this work, DSGN, a unified stochastic regularization framework designed to improve adversarial robustness, was proposed. By integrating input-dependent noise with geometric normalization, predictive uncertainty is effectively modeled and smoother decision boundaries are encouraged. Our results indicate that DSGN achieves a robust accuracy improvement of approximately 1% to 6% over the State of the arts baseline model under PGD and a 17% improvement under AutoAttack, demonstrating its effectiveness in enhancing adversarial robustness while maintaining high clean accuracy While the current results are encouraging, several promising directions remain for future research. These include extending the theoretical analysis to deeper, non-linear architectures to gain a deeper understanding of DSGN’s impact on generalization and the geometry of the loss landscape, designing adaptive noise mechanisms that dynamically adjust variance during training, and exploring the applicability of DSGN to alternative architectures such as Transformers and graph neural networks to assess its generalizability. Advancing these directions will further solidify the theoretical foundation of DSGN and broaden its practical utility across diverse real-world tasks. It is believed that this study offers a novel perspective on integrating stochastic and geometric principles for building reliable and robust deep learning systems.

## Figures and Tables

**Table 1:** Summary of major adversarial attack methods and their characteristics.

Attack Type	Method	Advantage	Limitation
White-Box	FGSM [5]	Extremely fast.	Weak perturbations.
	PGD [8]	Stable.	High computational cost.
	C&W [19]	Generates imperceptible examples.	Computationally intensive.
	AutoAttack [20]	Reliable parameter-free ensemble.	High resource requirements.
Black-Box	Query Methods [21,22]	No surrogate model needed.	Computationally expensive.
Black-Box	Transfer Methods [24,25]	High computational efficiency.	Effectiveness diminishes against robust targets.

**Table 2:** Comparison with noise injection techniques using ResNet-18 on CIFAR-10 and CIFAR-100.

Dataset	Method	Clean	FGSM	PGD^20^	C&W	MIFGSM	DeepFool	AutoAttack
	AT [54]	81.84	56.70	52.16	78.46	54.96	0.35	47.69
	Additive Noise [13]	81.24	59.19	57.61	80.84	57.83	73.44	62.25
	Multiplicative [47]	83.16	61.92	59.49	82.80	59.48	78.28	63.78
CIFAR-10	RPF [47]	83.79	62.71	61.27	83.60	60.72	79.43	64.38
	CTRW [48]	83.69	66.49	69.48	82.93	65.47	82.45	73.56
	RN [36]	79.47	74.61	74.55	79.35	74.46	–	67.07
	**DSGN**	**92.21**	**82.32**	**80.43**	**91.73**	**80.54**	**88.43**	**84.52**
	AT [47]	55.81	31.33	28.71	50.94	30.26	0.79	24.48
	Additive [13]	53.34	31.72	31.13	52.50	31.16	46.76	36.37
	Multiplicative [47]	54.52	34.09	32.58	54.61	32.45	50.90	38.13
CIFAR-100	RPF [47]	56.88	37.67	37.37	56.59	35.31	54.39	42.88
	CTRW [48]	56.63	38.85	42.01	56.32	38.20	55.56	45.58
	RN [36]	52.44	47.88	47.70	52.24	47.81	–	39.01
	**DSGN**	**67.71**	**50.23**	**48.23**	**66.32**	**49.36**	**65.32**	**56.34**

**Table 3:** Adversarial robustness evaluation of randomized techniques with WideResNet on CIFAR-10.

Method	Clean	FGSM	PGD^20^	MI-FGSM	AA
AT [54]	85.84	60.65	55.06	58.47	52.24
Random Bit [46]	–	57.95	53.96	56.32	53.30
Additive [13]	84.27	62.36	58.47	60.58	60.55
Multiplicative [47]	83.48	62.01	57.48	59.79	57.99
RPF [47]	86.49	63.95	63.73	60.77	68.71
CTRW [48]	–	71.34	66.72	65.99	74.23
RN [36]	84.95	82.31	82.06	82.10	69.32
**DSGN**	**92.53**	**83.42**	**83.31**	**82.28**	**84.92**

**Table 4:** Comparison with SOTA defense algorithms on CIFAR-10 and ImageNet.

Method	CIFAR-10		ImageNet	
	**PGD^20^**	**AA**	**PGD^10^**	**PGD^50^**
Additive Noise [13]	62.36	67.88	–	54.61
Overfit [54]	55.06	52.24	39.85	39.19
RobustWRN [49]	59.13	52.48	31.14	–
AWP [50]	58.14	54.04	–	–
Random Bit [46]	53.96	53.30	42.88	42.72
SAT [51]	56.01	51.83	–	42.30
LLR [52]	54.24	–	–	47.00
RPF [47]	63.71	68.71	56.56	55.41
CTRW [48]	71.34	74.35	59.29	60.16
RN [36]	82.06	69.32	55.93	43.89
**DSGN**	**83.31**	**84.92**	**74.26**	**72.27**

**Table 5:** Generalization study on different datasets. All results are reported using ResNet-18 as the backbone (except MNIST). Accuracy (%) under clean and adversarial (FGSM, PGD^20^) settings is shown.

**Model**	**MNIST**			**SVHN**			**CIFAR-10**		
	Clean	FGSM	PGD^20^	Clean	FGSM	PGD^20^	Clean	FGSM	PGD^20^
No Defense	**99.3**	33.5	18.1	**94.9**	18.6	5.9	**92.9**	21.3	2.3
**DSGN**	96.3	**91.8**	**61.3**	92.2	**83.2**	**80.4**	92.2	**82.3**	**80.4**
**Model**	**CIFAR-100**			**Tiny-ImageNet**			**Imagenette**		
	Clean	FGSM	PGD^20^	Clean	FGSM	PGD^20^	Clean	FGSM	PGD^20^
No Defense	**68.8**	12.8	1.5	**48.0**	9.0	1.0	**75.5**	9.5	0.0
**DSGN**	67.7	**50.2**	**48.2**	46.3	**23.4**	**16.4**	72.4	**31.8**	**20.2**

**Table 6:** Generalization study on different network architectures. Results show that our method consistently improves robustness across mainstream architectures.

**Model**	**VGG19**		**GoogLeNet**		**DenseNet121**	
	FGSM	PGD^20^	FGSM	PGD^20^	FGSM	PGD^20^
No Defense	16.2	3.7	14.6	1.4	13.9	0.2
**DSGN**	**62.3**	**51.2**	**78.2**	**64.6**	**75.1**	**66.8**
**Model**	**ResNet-32**		**ResNet-44**		**ResNet-56**	
	FGSM	PGD^20^	FGSM	PGD^20^	FGSM	PGD^20^
No Defense	11.4	0.0	19.5	1.3	19.7	1.2
**DSGN**	**60.4**	**53.2**	**66.1**	**62.8**	**72.2**	**69.1**

**Table 7:** Comparison of state-of-the-art methods for black-box n-Pixel attack on CIFAR-10 with a ResNet-18 backbone.

Attack Strength	AT	Additive Noise	Multiplicative	RPF	CTRM	RN	DSGN
Clean	81.4	81.2	83.1	83.7	83.6	79.4	92.2
1 pixel	68.6	66.2	63.1	68.8	75.3	76.4	84.4
2 pixels	64.6	63.1	60.1	64.1	71.7	75.9	81.4
3 pixels	59.7	54.3	53.9	58.7	65.1	63.2	75.0
5 pixels	—	—	48.3	—	—	50.0	—

## Data Availability

The data supporting the findings of this study are available and can be provided upon reasonable request.
